# Alpha 1-antitrypsin retention in an ectopic liver

**DOI:** 10.1186/1746-1596-6-16

**Published:** 2011-02-28

**Authors:** Matthias Dettmer, Gieri Cathomas, Niels Willi

**Affiliations:** 1 Cantonal Institute of Pathology, Liestal, Switzerland

## Abstract

Ectopic livers are infrequently reported in the literature. The reported size for ectopic livers range from a few millimeters up to several centimeters. They are often clinically silent and incidentally discovered during imaging of the hepatobiliary tract, regional surgical procedures or autopsy. They are predestined for benign liver diseases otherwise observed in normal livers like fatty change or develop malignancies such as hepatocellular carcinoma, in a manner analogous to the parent orthotopic liver. The presence of abnormal alpha 1-antitrypsin retention in an ectopic liver has, to our knowledge, not been reported in the literature. Hereby, we present the first reported case featuring alpha 1-antitrypsin retention in an ectopic liver attached to the fundus of the gallbladder and present the clinical, radiological and pathological findings in a caucasian woman undergoing cholecystectomy for acute cholecystitis. Special liver stains showed an alpha 1-antitrypsin retention which was confirmed immunohistochemically. Although ectopic livers are rare and usually an incidental finding, the radiologist and the surgeon should take this into the differential diagnosis of a mass attached to the gall bladder. A secondary disease should be considered by the pathologist in such a specimen and alpha 1-antitrypsin retention should be ruled out by special liver stains. Finally, such a finding should prompt the managing clinician to exclude systemic alpha-1 antitrypsin deficiency in the patient through further appropriate tests.

## Background

Ectopic liver is a rare finding in routine clinical imaging, during surgery or as a pathological specimen. The first case of an ectopic liver attached to the gallbladder was reported more than fifty years ago by Eisert and colleagues [1]. Occasionally, ectopic liver may cause clinical symptoms like abdominal pain due to recurrent torsion, compression of adjacent organs, intraperitoneal bleeding, obstruction of the esophagus or the portal vein, but usually, remains silent [2-4]. Most of the ectopic livers attached to the gallbladder are discovered during a routine cholecystectomy. Histologically, the ectopic liver tissue is usually normal but may show secondary changes associated with the underlying gallbladder pathology such as inflammatory alterations [5,6] or even malignant transformation to hepatocellular carcinoma [7,8]. We now report the first case of an alpha 1-antitrypsin retention in an ectopic liver suggestive of possible systemic alpha 1 anti-trypsin deficiency.

## Case presentation

**Figure 1:**
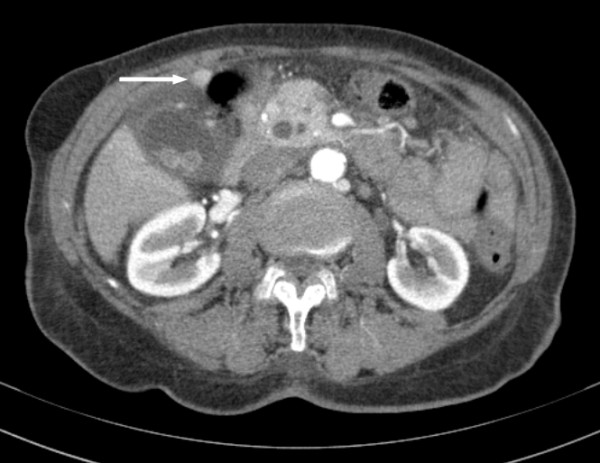
**Transversal CT scan, showing the ectopic liver (arrow)**.

**Figure 2:**
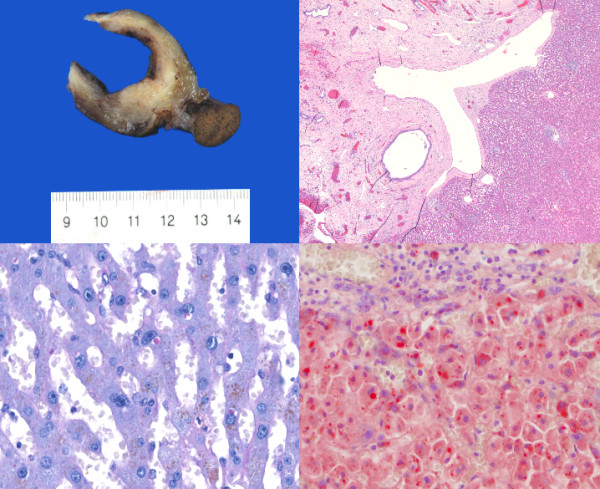
**Macroscopic and microscopic images**. Upper left: Macroscopic appearance. The ectopic liver is attached on the right side of the gall bladder, in the fundus. Upper right: Histologic appearance. The portal veins are easily identified in their y-appearance. Next to them the draining common bile duct (HE, 40×). Lower left: PAS positive and D-PAS resistant intracellular globules (D-PAS, 600×) Lower right: Immunhistochemistry for alpha1-antitrypsin shows multiple positive intracellular droplets (alpha1-antitrypsin, 600×)

A 91 year old previously healthy female patient underwent laparoscopic cholecystectomy four weeks after presenting with symptoms suggestive of acute cholecystitis. A preoperative abdominal computer tomography (CT) scan showed gall stones and a small mass of approximately 1.5 cm size attached to the fundus of the gall bladder (Figure 1). On macroscopic specimen examination, the mass had the color and appearance of normal liver tissue and contained portal tracts and a common bile duct which drained into the fundus of the gall bladder. Histologically, mild portal inflammation was observed due to the concomitant acute cholecystitis. No cholestasis or fibrosis was present. There were numerous periodic acid schiff (PAS) stained intracellular globules in the periportal hepatocytes which were also diastase resistant, raising the possibility that these globules contained abnormally retained alpha 1-antitrypsin. Further, immunohistochemical staining for alpha 1-antitrypsin was performed (Figure 2) using the Leica BOND-MAX™ fully automated immunohistochemical system. The antibody clone was ordered from Dako (Catalog# N1533). The dilution used was 1:16. Enzyme pretreatment for 5 minutes was performed to unmask epitopes (Enzyme 1, Leica, Catalog# AR9551). We used a Leica detection kit (Leica Bond polymer refine red detection, Catalog# DS9390). The biotin free compact antibody polymer labeled with alkaline phosphatase is highly specific and sensitive. Antigen retrieval was not done. All procedures were performed according to manufacturer's protocols.

## Discussion

Ectopic livers attached to the gallbladder are very rare in clinical practice, and have been estimated to occur in 0.23 to 0.47% of excised gallbladders. It was first described by Eisert in 1940 with an incidence of 0.23%; in another more recent laparoscopic case series, an incidence of 0.47% was noted [1,3]. Based on the relationship to the orthotopic liver, one may classify heterotopic liver tissue as accessory livers when they are connected to the parent liver by a thin stalk, or as true ectopic livers when no such connection can be demonstrated [2]. The most common site for ectopic liver is the serosal surface of the gallbladder. Such ectopic livers are known to form nodules ranging from several millimeters to several centimeters in size [3,9].

The increasing incidence of ectopic liver excisions is most likely the result of more thorough clinical and pathological work-up [3-6].

Several pathogenetic mechanisms for the development of liver heterotopias have been postulated. During human embryogenesis, there is a very proximate relationship between the developing liver, the duodenum and the gall bladder. A migration or displacement of a cranial part of the liver bud during embryogenesis seems the most likely explanation for ectopic livers and would explain their localization within structures associated with organogenesis of the liver, including the gall bladder, the gastrohepatic ligament, the adrenal glands, pancreas and the splenic capsule. Ectopic livers are rarely found in an intrathoracic location, such as attached to the surface of the heart or the pericardium. Others hypotheses of origin include the possibility that they occur as developmental anomalies due to atrophy or regression of the connecting tissue to the parent liver [1,10-17]. Interestingly, ectopic liver tissue is not always as fully developed as the parent liver and major structures such as the main bile duct or important vascular structures such as the main portal vein or hepatic artery branch may be absent. This compromise in direct blood supply has been postulated to account for their increased susceptibility to injury and malignant change [8,18].

The surgeon and the radiologist would be advised to consider the possibility of ectopic liver in addition to edema, inflammation, cholesterol polyps, adenomyomatosis, adenoma and primary or secondary malignancies in the differential diagnosis of a patient presenting with thickening of the gall bladder wall or a suspicious mass lesion [10,19]. On a CT scan, the mass would be isointense to normal liver tissue. Ectopic liver is also histologically often identical to *bona fide *liver tissue or may show identical pathological changes seen as normal orthotopic liver. Fatty infiltration, alcoholic cirrhosis, haemosiderosis and primary hepatocellular carcinoma have been reported in ectopic liver, along with secondary malignancy [3,7,8,20,21]. For the first time, we report a case of alpha 1-antitrypsin retention with radiologic correlation, and macroscopic and microscopic images in an ectopic liver, adding to the list of pathological abnormalities that may be seen in an ectopic liver.

Clinically, alpha 1-antitrypsin deficiency usually presents with dyspnea that may resemble an asthmatic exacerbation, which does not respond to standard anti asthmatic treatment. Patients often develop severe emphysema by their early thirties and forties, in the absence of a history of smoking. Liver impairment progressing to liver failure or cirrhosis has been attributed to the toxic accumulation of synthesized mutant alpha 1-antitrypsin peptide within the cytoplasm of the hepatocytes. The disease may be frequently under recognized and underdiagnosed, as many patients may be clinically silent [22]. Typically, alpha 1-antitrypsin deficiency is diagnosed by the measurement of serum or plasma protein levels, alpha 1-antitrypsin protein phenotyping of serum or plasma or by alpha 1-antitrypsin genotyping [23].

In this case, the patient did not have any history of liver or lung disease, nor did she display any symptoms related to alpha 1-antitrypsin deficiency. The patient declined further confimatory blood tests for alpha 1-antitrypsin in view of her advanced age and this matter was not further pursued by the managing clinician.

Nonetheless, we strongly believe that alpha 1-antitrypsin accumulation diagnosed histologically with PAS/D-PAS and confirmed by immunohistochemical staining for alpha 1 anti-trypsin should prompt the treating clinician to perform a work-up for alpha 1-antitrypsin deficiency. In fact, a case of clinically silent alpha 1-antitrypsin deficiency, diagnosed in an orthotopic liver has been described in a previous case report [24].

However, ectopic livers are prone to all kinds of pathologic changes. This could account for the possibility that the observed intracellular alpha-1 antitrypsin retention might be unrelated to a genuine alpha 1 antitrypsin deficiency genotype and represent only a local phenomen.

## Conclusion

The possibility of ectopic liver has to be considered in the differential diagnosis of a gall bladder wall thickening or mass. A thorough examination of ectopic liver tissue by the pathologist may uncover clues to a systemic disease, such alpha 1 antitrypsin deficiency.

## Consent

Written informed consent was obtained from the patient for publication of this case report and accompanying images. A copy of the written consent is available for review by the Editor-in-Chief of Diagnostic Pathology.

## Competing interests

The authors declare that they have no competing interests.

## Authors' contributions

All authors contributed to this article. All authors read and approved the final manuscript.
